# Transcriptome profiling reveals differential gene expression of detoxification enzymes in a hemimetabolous tobacco pest after feeding on jasmonate-silenced *Nicotiana attenuata* plants

**DOI:** 10.1186/s12864-016-3348-0

**Published:** 2016-12-08

**Authors:** Cristina M. Crava, Christoph Brütting, Ian T. Baldwin

**Affiliations:** 1 Department of Molecular Ecology, Max Planck Institute for Chemical Ecology, Hans-Knoell strasse 8, D-07745 Jena, Germany; 2 Present Address: Department of Sustainable Ecosystems and Bio-resources, Research and Innovation Centre, Fondazione Edmund Mach, via Mach 1, 38010 San Michele all’Adige, Italy

**Keywords:** Coyote tobacco, Trophic interactions, Piercing-sucking herbivory, Detoxification, Heteroptera

## Abstract

**Background:**

The evolutionary arms race between plants and insects has driven the co-evolution of sophisticated defense mechanisms used by plants to deter herbivores and equally sophisticated strategies that enable phytophagous insects to rapidly detoxify the plant’s defense metabolites. In this study, we identify the genetic determinants that enable the mirid, *Tupiocoris notatus,* to feed on its well-defended host plant, *Nicotiana attenuata*, an outstanding model for plant-insect interaction studies.

**Results:**

We used an RNAseq approach to evaluate the global gene expression of *T. notatus* after feeding on a transgenic *N. attenuata* line which does not accumulate jasmonic acid (JA) after herbivory, and consequently accumulates very low levels of defense metabolites. Using Illumina sequencing, we generated a *de novo* assembled transcriptome which resulted in 63,062 contigs (putative transcript isoforms) contained in 42,610 isotigs (putative identified genes). Differential expression analysis based on RSEM-estimated transcript abundances identified 82 differentially expressed (DE) transcripts between *T. notatus* fed on wild-type and the defenseless plants. The same analysis conducted with Corset-estimated transcript abundances identified 59 DE clusters containing 85 transcripts. In both analyses, a larger number of DE transcripts were found down-regulated in mirids feeding on JA-silenced plants (around 70%). Among these down-regulated transcripts we identified seven transcripts possibly involved in the detoxification of *N. attenuata* defense metabolite, specifically, one glutathione-S-transferase (GST), one UDP-glucosyltransferase (UGT), five cytochrome P450 (P450s), and six serine proteases. Real-time quantitative PCR confirmed the down-regulation for six transcripts (encoding GST, UGT and four P450s) and revealed that their expression was only slightly decreased in mirids feeding on another *N. attenuata* transgenic line specifically silenced in the accumulation of diterpene glycosides, one of the many classes of JA-mediated defenses in *N. attenuata*.

**Conclusions:**

The results provide a transcriptional overview of the changes in a specialist hemimetabolous insect associated with feeding on host plants depleted in chemical defenses. Overall, the analysis reveals that *T. notatus* responses to host plant defenses are narrow and engages P450 detoxification pathways. It further identifies candidate genes which can be tested in future experiments to understand their role in shaping the *T. notatus-N. attenuata* interaction.

**Electronic supplementary material:**

The online version of this article (doi:10.1186/s12864-016-3348-0) contains supplementary material, which is available to authorized users.

## Background

Plants are constantly challenged by insect herbivores, but they defend themselves with a vast array of chemical defenses, which span from direct defenses, such as toxins or feeding deterrents that diminish plant palatability, to indirect defenses such as the emission of volatile compounds that attract parasitoids and predators of herbivores [1]. Consistent exposure to toxic or anti-digestive compounds has selected for adaptions in herbivorous insects to the defenses of host plants with the result that well-defended plants have become targets of herbivory. This co-evolutionary process has to a large extent shaped the host plant specialization in insects [2] and the majority of phytophagous insects feed only on a small number of host plants [3]. The most common evolutionary innovations that enable insects to reliably cope with noxious metabolites produced by their host plants can be classified in three main categories of biochemical strategies: mechanisms that result in enzymatic detoxification, rapid excretion and metabolic changes that allow for the sequestration of plant toxins for the insects own defense [4, 5].

Enzymatic detoxification of ingested plant defenses is commonly used by both generalist and specialist insect herbivores [3]. Enzymes that are involved in xenobiotic detoxification frequently belong to the large cytochrome P450 monooxigenase (P450) family which catalyze a wide range of reactions in animal and plants [6, 7]. The best-studied examples of the role of P450s in plant metabolite detoxification come from different lepidopteran families. Several *Papilio* species as well as the parsnip webworm, *Depressaria pastinacella*, use a CYP6B enzyme to detoxify toxic fouranocoumarins produced by their host plants [8–10]. In the navel orangeworn, *Amyelois transitella*, a closely related CYP6AB11 metabolizes imperatorin [11] and in the generalist *Helicoverpa zea*, CYP6B8 metabolizes a variety of plant compounds, including several phenolics and flavonoids [12]. *Helicoverpa armigera* requires the activity of CYP6AE14 to develop on cotton containing gossypol [13], and another cotton pest belonging to a different order, the aphid *Aphis gossypii*, relies on a similar enzyme (CYP6DA2) to deal with the same toxin [14]. Examples of detoxification carried out by P450 enzymes are also present among Coleoptera [15], Diptera [16] and Hymenoptera [17]. In addition to the direct evidence of detoxification provided by the above examples, P450s have also been associated with resistance to plant secondary metabolites in a number of other species, including *Manduca sexta*, *Drosophila* species from the Sonora desert and mosquito larvae, all of which have been shown to increase the accumulation of transcripts coding for specific P450s in response to the ingestion of defense compounds (reviewed in [7]).

Another common detoxification strategy, which is often a subsequent step to P450-mediated functionalization reactions, involves the addition of sugars or glutathione which frequently reduces the reactivity of functional groups and increases their water solubility making the toxins easier to excrete. Glutathione S-transferases (GSTs) and UDP-glucosyltransferases (UGTs) are examples of such enzymes. In *Spodoptera* spp., larvae feeding on maize, which produces the toxic aglucone DIMBOA when attacked, detoxify the toxin in their guts by the addition of glucose [18]. *Helicoverpa* larvae uses an UGT to glycosylate the alkaloid capsaicin produced by pepper fruits [19] and *M. sexta* larvae glycosylate several toxic plant phenolics produced by its host plants [20]. *M. sexta* larvae are also one of the likely numerous examples in which host plant specialization has enabled them to exploit plant metabolites for their own defense against predators. While feeding on nicotine-containing *Nicotiana* species, *M. sexta* larvae pass the majority of the nicotine they ingest through with their frass but can exhale a small amount through their spiracles in response to attack from spiders. A largely mid-gut expressed P450, CYP6B46 is required for this unusual defensive co-option of an otherwise excreted defense metabolite [21]. To date, the ability to sequester plant secondary metabolites has been found in more than 250 insect families and in many cases, the sequestration has been shown to increase insect defenses [22].

Attacked plants not only produce toxic secondary metabolites but also proteinaceous effectors such as proteinase inhibitors (PIs). PIs interfere with insect digestion processes and reduce the availability of essential amino acids required for growth, development and reproduction [23]. Insects overcome the deleterious effects of ingesting PIs by either the direct hydrolysis of PIs with specific proteinases and/or transcriptionally upregulating the production of PI-insensitive isoforms or over-producing sensitive proteinases [24–26].

The majority of examples of insect adaptation to harmful plant metabolites comes from three orders: Lepidoptera, Coleoptera or Diptera. Very few examples come from hemimetabolous insects, such for Hemiptera, the so-called plant bugs. *Tupiocoris notatus* Distant (Hemiptera: Miridae) is a cell-content feeder bug with a wide distribution in the southern United States. It specializes on solanaceous species, attacking both wild plants and cultivated crops. Its main hosts are species from *Nicotiana* and *Datura* families [27]. In the Great Basin Desert of the southwestern USA, *T. notatus* attacks the native tobacco plant *Nicotiana attenuata* (Torr. Ex Watson), a model species for plant-insect interactions. *N. attenuata*’s response to insect attack involves a well-characterized signaling system which leads to the accumulation of toxic secondary metabolites such as nicotine, phenolic compounds or diterpeneglycosides (DTGs), defensive proteins like trypsin PIs (TPIs) and volatile organic compounds (VOCs) [28–32]. When the attacker is the lepidopteran *M. sexta*, *N. attenuata’s* responses are elicited by fatty acid-amino acid conjugates from caterpillar saliva that are introduced into plant wounds during feeding [33]. Recognition of these elicitors is followed by a jasmonic acid (JA) burst that mediates the accumulation of defense metabolites [34]. How *N. attenuata* recognizes herbivory from the important piercing-sucking herbivore *T. notatus* remains unknown; however the extensive tissue damage produced by mirid feeding elicits a very similar suite of responses as those elicited by *M. sexta*, which includes increased concentrations of phenolics, DTGs and TPIs [35]. Similarly, the mechanisms that allow *T. notatus* to cope with the array of toxic metabolites produced by *N. attenuata* remain unexplored. Previous field experiments on *T. notatus* preferences among different *N. attenuata* transgenic lines silenced in several layers of defenses revealed that *T. notatus* feeds with apparent impunity on its host plant since it even prefers better defended wild-type plants than those impaired in JA biosynthesis; the opposite occurs for most other herbivores that attack *N. attenuata* [36]. This has led to the hypothesis that *T. notatus* might be even more adapted to *N. attenuata* defense metabolites than other generalist herbivores, such as *Empoasca* spp. [36]. However, when single defense compounds were silenced, both DTGs [32, 37] and TPIs [38] emerged as clear determinants of *T. notatus* host choice.

**Fig. 1:**
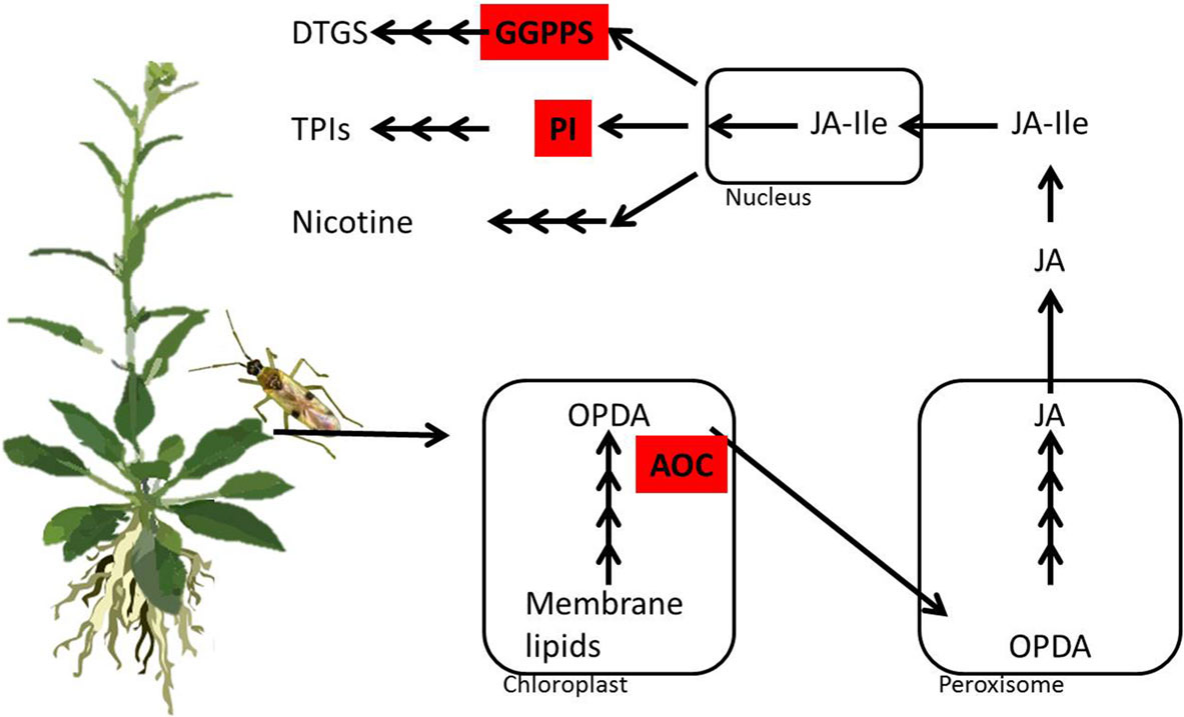
Overview of the transformed plants used to dissect the mechanisms used by *Tupiocoris notatus* to tolerate toxic metabolites produced by its host plant, *Nicotiana attenuata*. Schematic of the signaling and biosynthetic pathways of jasmonate-induced defenses in *N. attenuata*. The enzymes silenced in the transgenic RNAi lines are highlighted in red font. Abbreviations: OPDA, 12-oxophytodienoic acid; AOC, allene oxide cyclase; JA, jasmonic acid, JA-Ile, jasmonic acid-isoleucine; GGPPS, geranyl diphosphate synthase; DTGs, diterpene glycosides; PI, proteinase inhibitors; TPIs, trypsin proteinase inhibitors

To understand *T. notatus’s* responses to *N. attenuata* defenses, we used next-generation sequencing and a *N. attenuata* transgenic line deficient in JA biosynthesis (Fig. 1). Allene oxide cyclase (AOC) is a key enzyme in JA production, mediating the last step of 12-oxo-phytodienoic acid (OPDA) biosynthesis. Plants silenced in this gene do not accumulate most of the known defense metabolites produced by the plant [39], and hence are ideal for identifying candidate resistance genes that help *T. notatus* exploit *N. attenuata* as a host. We hypothesized that *T. notatus* feeding on JA-deficient plant would have a decreased expression of genes used in the detoxification of toxic metabolites. We then used transgenic lines specifically silenced in DTGs and TPIs accumulation to evaluate if the expression of particular candidate genes were involved in the detoxification of these two groups of defenses which are important in determining the host choice of mirids.

## Methods

### Plant and insect material

We used previously characterized homozygous, inverted-repeat (ir) RNAi transformed lines of the second generation which were silenced in JA biosynthesis (ir*AOC*, line number A-07-457-1; [39]), TPI activity (ir*PI*, line number A-04-186-1; [23]) and DTGs accumulation (ir*GGPPS*, line number A-08-230-2; [32]) (Fig. 1). An empty-vector (EV) transformed plant line (A-04-266-3) was used as controls. Seed germination and glasshouse growth conditions are described in [40]. Briefly, seeds were germinated on sterile Gamborg B5 medium (Duchefa) after 1 h of treatment with diluted smoke (House of Herbs) and 1 mM GA3 (Roth). Ten days after germination, seedlings were transferred into Teku pots containing a peat-based substrate, and after 10–12 d plantlets were transplanted into individual 2-L pots with the same substrate. In the glasshouse, plants were grown at 24 °C to 26 °C, relative humidity ~60%, and supplemented with a light:dark regime of 16:8 h.

*T. notatus* were from a colony started with insects collected in the Utah desert around our field station (Lytle Ranch Preserve, Washington County, Utah, USA). The colony was maintained in a glass-cage under glasshouse conditions, and fed hydroponically cultivated *N. attenuata* plants, which were refreshed once or twice per week depending on the feed damage caused by the mirids.

### RNA sample preparation and sequencing

Approximately 150–200 *T. notatus* were confined on either an EV or an ir*AOC* plant in an insect rearing cage (60 × 60 × 180 cm, BugDorms) for 3 days. After collection, nymphs were separated from adults and 80 adults were used for each replicate, and three replicates per treatment were harvested. Total RNA extraction was performed with TRIzol reagent (Invitrogen) according to the manufacturer’s instructions. Poly-A RNA purification, library preparation and Illumina sequencing were carried out at the BGI sequencing facilities (Hong-Kong). The six libraries were sequenced in a single lane in a HiSeq 2000 machine (Illumina), to obtain 100 bp paired-end reads. Adapter sequences and low quality sequences were removed using Trimmomatic to obtain high quality (HQ) reads [41]. Orphan reads were collated in a separate file and used as singletons during the *de novo* assembly. The data sets are available at the NCBI Short Read Archive (SRA) with accession number PRJNA343704.

### *De novo* assembly of the *T. notatus* transcriptome

*De novo* assembly of a *T. notatus* reference transcriptome was performed using short read assembler Trinity with default parameters, using *in silico* normalization and min_kmer_cov 2 options [42]. Trinity output delivers two output types: contigs, which represent putative transcript isoforms, and isotigs, which represent putatively identified genes. HQ reads were then mapped back to the assembled transcriptome using Bowtie [43], and mapping statistics were estimated by RSEM [44]. Finally, contigs with less than one IsoPct (percentage of expression for a given contig compared with all expression from a particular isotig) or less than 0.3 fragments per kilobase transcript length per million fragments mapped (FPKM) were discarded.

We employed two different assembly strategies to cope with the high heterozygosity of the samples. In the first strategy (ALL), we assembled all HQ reads obtained from sequencing (derived from 480 insects), and in the second one (TWO) we only used HQ reads from two replicates per treatment (320 insects in total). These two transcriptomes were then compared in terms of lowest total number of contigs, highest number of mapped HQ reads, highest number of uniquely HQ mapped reads, highest number of unique Blastx hits against *Acyrthosiphon pisum* proteome and ortholog hit ratio (OHR) [45]. According to these parameters, the best assembled transcriptome was then used as a reference transcriptome, and has been deposited at GenBank under the accession GFBA00000000. The version described in this paper is the first version, GFBA01000000.

### Annotation

Annotation of the reference transcriptome was conducted on a local server against the National Center for Biotechnology Information (NCBI) non-redundant database (nr), Swissprot database and Uniref90 database using Blastx with an e-value of 10^−5^. Additional Blastx search comparison was performed in order to obtain a set of putative orthologs from *A. pisum*, *Diaphorina citri* and *Drosophila melanogaster* proteomes. Gene ontology (GO) terms were retrieved by using Blast2GO software [46] with default parameters, using Blastx results against a local nr database. GO annotation resulting from Blast2GO was implemented by running InterProScan and merging the results. Finally, the GO-slim function was used to summarize annotation results. Enzyme classification codes (EC) and metabolic pathway annotation (KEGG, Kyoto Encyclopedia of Genes and Genomes) were generated by Blast2GO from direct mapping of GO terms to their enzyme code equivalents.

### Differential gene expression analysis

To obtain the count matrix that contains the number of reads mapped to each contig in each sample, we used two different methods to estimate counts in case of multi-mapping reads. First, a SAM file was generated per each sample using Bowtie allowing multi-mapping. Then, in the first approach the SAM files were fed to RSEM implemented in the Trinity pipeline. In case of multi-mapping reads, RSEM employs a “rescue” method based on estimated maximum likelihood expression levels, which allows partitioning and distributing portions of a multiread’s expression value among all of the contigs to which it maps. When RSEM is run with Trinity-assembled transcriptomes, it delivers two outputs: estimated counts at isoform (contig) level and at the gene (isotig) level. In parallel, SAM files were also processed by Corset [47], which hierarchically clusters the contigs based on the proportion of shared reads and expression patterns. All the reads are uniquely assigned to a cluster; hence, each read is only counted once, even though the reads were originally multi-mapped to different contigs. Matrix counts estimated by the two methods were then used to identify differentially-expressed (DE) contigs through EdgeR [48] in R using RStudio. Heatmaps showing the expression level of specific contigs were generated in R using heatmap.2 implemented in the package gPlots.

### Phylogenetic analyses of CYP450, GST and UGT families

Contigs annotated as P450s, GSTs or UGTs after Blastx were selected for phylogenetic analysis. First, contigs belonging to the same isotig were aligned with the software BioEdit 7.2.5 [49], alignments were manually inspected and the longest contig was selected. Secondly, coding sequences (CDS) (either complete or partial) were identified. For each gene family, an alignment of all *T. notatus* CDS was generated by TranslatorX [50] using the Muscle algorithm [51], and a maximum likelihood (ML) tree was constructed in MEGA v6 [52]. Redundant sequences were then merged to obtain a non-redundant list of putatively unique transcripts (PUTs), although it should be noted that we cannot exclude that some PUTs might be allelic variants of the same gene. PUTs were then translated into proteins and for each gene family, an amino acid alignment was constructed in MEGA v6 using genomically annotated sequences from other insect species: *A. pisum* [53] and *Rhodnius prolixus* P450s and GSTs [54], *Bombyx mori* [19] and *D. melanogaster* UGTs [55]. The alignments were manually edited and used to construct ML trees in MEGA v6 using 100 bootstrap pseudo-replicates.

### Real- time quantitative PCR

*T. notatus* adults were reared on EV, ir*AOC*, ir*GGPPS* or ir*PI N. attenuata* plants for 3 days in insect cages as described above. We used 3 cages for each genotype, and each cage served as one replicate. RNA was extracted from 80 *T. notatus* adults for each sample with TRIzol (Invitrogen), according to the manufacturer instructions. RNA quality was checked with an Agilent 2100 Bioanalyzer and DNase-treatment was done using TURBO DNA-free Kit (Ambion). cDNA was synthesized by reverse transcription using oligo(dT) primer and RevertAid reverse transcriptase (Invitrogen) with 0.5 μg of template RNA. Real time quantitative PCR (RT-qPCR) was performed using ribosomal protein 28S and alpha tubulin reference genes. RT-qPCR was carried out on a Stratagene M × 3005P machine using a Taykon NO ROX SYBR Master Mix dTTP Blue (Takyon) with ROX as reference dye. The primer sequences are provided in Additional file 1: Table S1. Expression levels were quantified through the Pfaffl method [56] using normalization against the geometric mean of the housekeeping gene expression [57].

## Results

### *De novo* transcriptome assembly and functional annotation of *T. notatus* transcriptome

**Fig. 2:**
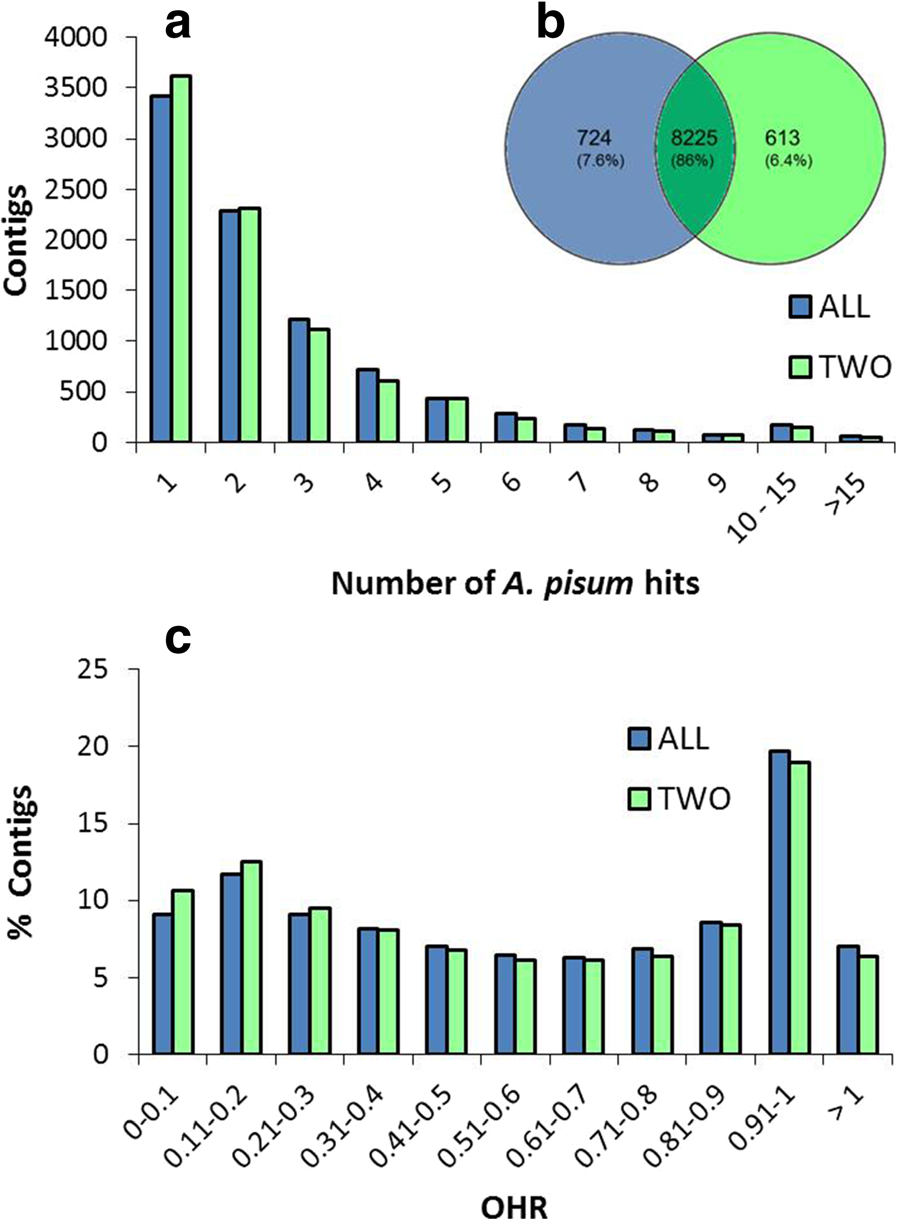
Quality assessment of the two *de novo* assembled *Tupiocoris notatus* transcriptomes. Each transcriptome was assembled using the full set of HQ reads (ALL) or using two thirds of HQ reads (TWO). **a** Frequency distribution of the number of *T. notatus* contigs that hit a single A*cyrthosiphon pisum* sequence after Blastx; **b** Venn diagram showing the number of *A. pisum* sequences hit by at least one *T. notatus* contig from each transcriptome; **c** Overall distribution of ortholog hit ratio (OHR) calculated using Blastx annotation against the *A. pisum* proteome

Ilumina sequencing delivered 66 M 100-bp paired-end reads. On average, the same numbers of reads (around 33 M) were obtained from each treatment: whole *T. notatus* feeding on EV and ir*AOC* defense-less plants (Table 1). After trimming, *de novo* transcriptome assembly was carried out in parallel using two different strategies. Since our samples consisted of eighty individuals per replicate, they might display a high heterozygosity that would affect *de novo* assembly and subsequent differential expression analysis. We assembled in parallel a transcriptome from a smaller subset of HQ reads (transcriptome TWO), and we then compared its statistics with those of a transcriptome assembled using all HQ reads (transcriptome ALL). In trascriptome TWO, the number of contigs obtained decreased from 71,182 to 63,062 whereas the N50 decreased from 1,891 to 1,746 (Table 2). Interestingly, transcriptome TWO was mapped by the same quantity of HQ reads as did transcriptome ALL despite its fewer contigs, and the number of uniquely HQ mapping reads increased from 58 to 62% (Table 2). Thus, the decreased number of contigs in transcriptome TWO was likely due to a better assembly and a consequent reduction of redundant isoforms. Blastx results against the pea aphid proteome (which consisted of 23,090 predicted proteins) were consistent with the results: 37 and 35% of the contigs from transcriptomes TWO and ALL found matches, respectively (Table 2, Fig. 2b), and a higher number of *A. pisum* sequences were matched once by contigs from transcriptome TWO (3,615) compared to those from transcriptome ALL (3,423) (Fig. 2a), consistent with a decrease in redundancy for transcriptome TWO. Blastx results were also used to calculate the ortholog hit ratio (OHR) for both transcriptomes, which estimates the completeness of a transcript contained in each assembled contig [45] (Fig. 2c). Transcriptome ALL showed an OHR of 0.57, slighter higher than transcriptome TWO (0.55). In short, the assembly derived from transcriptome TWO had fewer isoforms and lower redundancy without a drastic decrease in contig size or completeness, and hence was used as the reference transcriptome.

**Table 1:** Sequencing statistics

	*T. notatus* feeding on EV plants			*T. notatus* feeding on *ir*AOC plants			Total
	r1	r2	r3	r1	r2	r3	
Total reads	10′747′823	10′873′406	11′717′762	10′844′053	10′846′457	109′245′20	65′954′021
Paired HQ reads	9′675′776	9′820′575	10′555′522	9′725′240	9′802′804	9′896′331	59′476′248
Orphan HQ reads	888′077	874′135	969′082	934′701	869′226	853′373	5′388′594

**Table 2:** Transcriptome statistics

	ALL	TWO
Number of reads used for assembly	64′864′842	42′590′534
Number of contigs (Putative transcript isoforms)	71′182	63′062
Number of isotigs (Putative identified genes)	45′629	42′610
N50 (bp)	1′891	1′746
Maximum length	20′032	15′025
Minimum length	224	224
% of reads mapped to the transcriptome	81.7	82.0
% of uniquely mapping reads	58.2	61.5
Average ortholog hit ratio	0.57	0.55
Total Blastx hits against *A. pisum* proteome	24′936 (35.0%)	23′173 (36.7%)

**Fig. 3:**
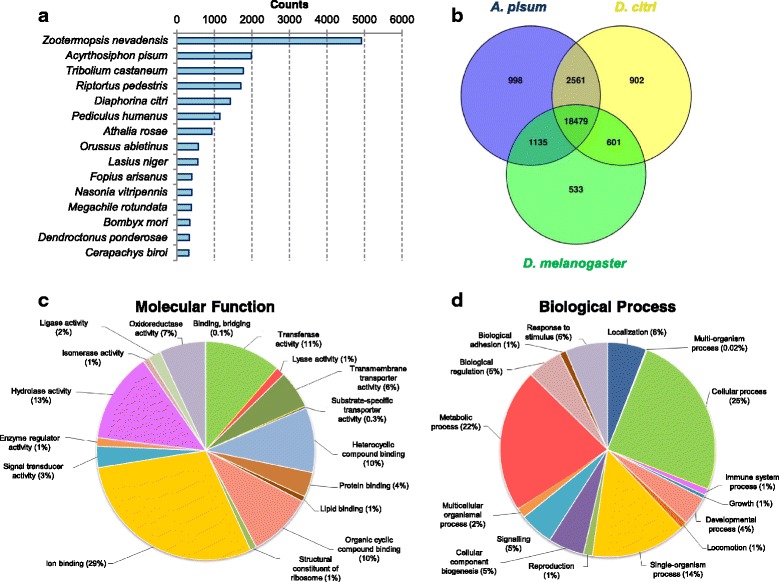
Annotation of *Tupiocoris notatus* transcriptome. **a** Species distribution of the top Blastx hit performed against NCBI nr database; **b** Venn diagram showing the number of orthologous genes shared between *T. notatus*, *Acyrthosiphon pisum*, *Diaphorina citri* and *Drosophila melanogaster*; **c** and **d**) Gene Onthology (GO) assignments as predicted by Blast2GO at GO level 3 and 2 for the categories Molecular Function and Biological Process

The reference transcriptome was used as the query in Blastx searches (e- value 10^−5^) against different protein databases. Roughly 41% of the contigs matched sequences present in the nr database, 33% sequences in the Swissprot database and 47% in the Uniref90 database. More than half of the contigs (33,258) had no blast result in any of the databases, suggesting a high number of orphan genes that may be genus- or species-specific. Among the Blastx results against nr database, most of the contigs were identified as homologs of species within Insecta (89%) (Fig. 3a). Moreover, 125 hits corresponded to bacteria (in particular, 11 hits to *Wolbachia* sp.) and 23 hits to viruses. We then analyzed the part of the contigs which had counterparts in other insect species, using Blastx searches against the *A. pisum*, *D. citri* and *D. melanogaster* proteomes. The Venn diagram in Fig. 3b shows the distribution of hits above the e-value threshold of 10^−5^. We observed that the largest number of hits were obtained from blasting against the two hemipteran species: the pea aphid *A. pisum* (37%) and the psyllid *D. citri* (36%), whereas hits resulting from blasting against *D. melanogaster* proteome covered 33% the transcriptome.

Gene Ontology (GO) classification was used to functionally analyze the *T. notatus* reference transcriptome. GO annotation associates analyzed transcripts with terms from hierarchical vocabularies describing molecular function, cellular localization or biological process. Of 27,092 contigs with similarity in Blastx search, 18,493 (70%) could be functionally annotated. Fig. 3c and d show the Biological Process GO-level 2 and Molecular Function GO-level 3 classes in which *T. notatus* contigs were classified. The most prominent Biological Process categories were related to basic cell functions, such as cellular process (25%), metabolic process (25%) and single-organism process (14%). Among the most prominent GO terms retrieved in Molecular Function, strong representation of ion binding (29%), followed by terms associated to enzymatic activity like hydrolase activity (13%) and transferase activity (11%) were found. Blast2GO was used to mine the Kyoto Encyclopedia of Genes and Genomes (KEGG) database to identify potential pathways represented in the transcriptome. A total of 6,537 contigs were mapped to 126 KEGGS pathways. The largest number of contigs was annotated as biosynthesis of secondary metabolites, purine metabolism, amino sugar and nucleotide sugar metabolism (Additional file 2: Figure S1).

### Expression of transcripts encoding detoxification genes decreases in *T. notatus* fed on defense-less plants

**Fig. 4:**
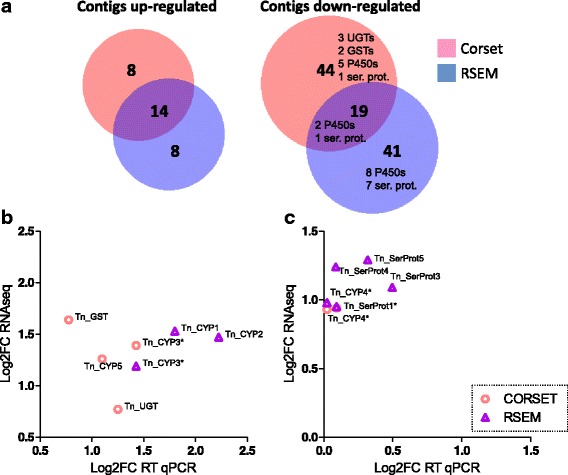
Comparison between results obtained with Corset- or RSEM-estimated transcript abundances, and RT-qPCR. **a** Venn diagrams depicting the number of up- and down-regulated contigs in mirids feeding on JA-silenced *Nicotiana attenuata* plants identified by RNAseq analysis. Among the down-regulated genes, a list of differentially-expressed (DE) contigs annotated as detoxification enzymes or serine proteases (ser. prot) is shown; **b**) and **c**) Scatter plots showing the correlation between log2 fold-change (log2FC) estimated by RNAseq or by RT-qPCR for putatively unique transcripts (PUTs) likely involved in *N. attenuata* metabolite detoxification. Asterisks indicate contigs that have been identified as DE by RNAseq analysis using both Corset- or RSEM-estimated transcript abundances

We determined contigs that were differentially expressed (DE) in *T. notatus* in response to feeding on JA-silenced ir*AOC* plants by using two different methods for transcript clustering and abundance estimations. Both methods revealed significant differential expression of only few contigs at the False Discovery Rate (FDR) of < 0.05 (Fig. 4a, Additional file 2: Figure S2): DE isotigs identified using transcript abundances estimated by RSEM were 45 (for a total of 82 contigs) whereas Corset transcript quantification led to the identification of 59 clusters (for a total of 85 contigs) (Additional file 3: Table S2, Additional file 4: Table S3).

Among the results obtained with RSEM-estimated transcript abundances, 12 isotigs (containing 22 contigs) were up-regulated (27%) in insects feeding on ir*AOC* plants whereas 33 (containing 60 contigs) were down-regulated (73%). Overall, 19 DE isotigs contained contigs with no blast or uninformative annotations. Among down-regulated isotigs, nine contained contigs annotated as enzymes possibly related to detoxification of *N. attenuata* defense metabolites, namely eight P450s and six serine proteases (Additional file 3: Table S2, Fig. 4a). In contrast, contigs identified as up-regulated during feeding on ir*AOC* plants were most likely involved in primary metabolism.

Among results obtained with Corset-estimated abundances, 15 clusters (containing 22 contigs) were up-regulated (26%) whereas 44 (containing 63 contigs) were down-regulated (74%). 31 DE Corset clusters contained contigs with uninformative or no blast hits. Among clusters down-regulated in mirids feeding on ir*AOC* plants, seven contained contigs that might be involved in *N. attenuata* secondary metabolite detoxification, since five contigs were annotated as P450s, three as UGTs and two as GSTs. Also, a contig annotated as serine protease was down-regulated (Additional file 4: Table S3, Fig. 4a). On the contrary, up-regulated contigs likely belonged to primary metabolism.

When the results from both methods were compared, we found 33 contigs that were commonly identified by both methods. Of these, 14 were up-regulated and 19 down-regulated (Fig. 4a). Among the down-regulated contigs, two were annotated as P450s and one as a serine protease; for these, fold-change values obtained by EdgeR using RSEM-estimated or Corset-estimated count matrix were highly similar (Fig. 4b and c).

### Expression patterns of detoxification genes in mirids feeding on different transgenic lines silenced in different defense mechanisms

Contigs encoding P450s, GSTs, UGTs or serine proteases, which were down-regulated in mirids feeding on ir*AOC* plants and thus suspected to play a role in *N. attenuata* defense metabolite response, were manually inspected and merged to obtain putatively unique transcripts (PUT). Complete CDS were obtained for both a GST and a UGT, as well as for six serine proteases (Table S1). In the case of P450s, down-regulated contigs were assembled in five PUTs, of which only one contained a complete CDS. The remaining four PUTs contained fragments of CDS and we cannot rule out that some of these may belong to the same gene.

**Fig. 5:**
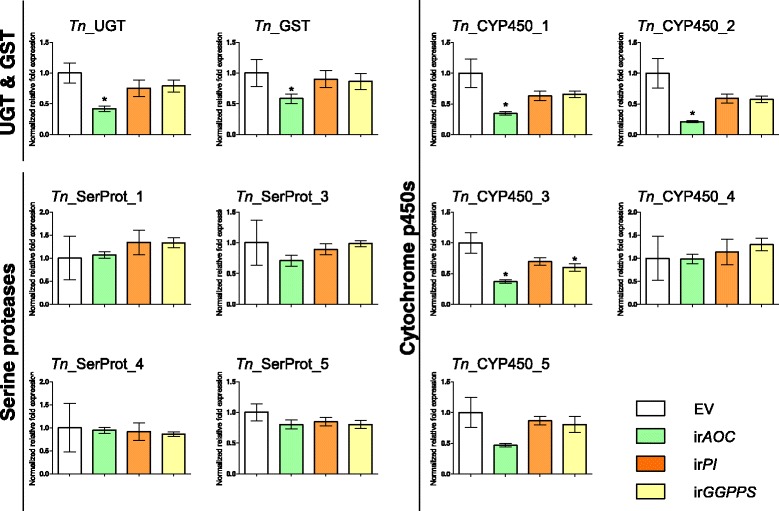
Expression of putatively unique transcripts (PUTs) involved in response to *Nicotiana attenuata* 's jasmonic acid-mediated defenses in *Tupiocoris notatus* feeding on different transgenic lines with reduced degrees of defenses. Expression was checked by RT-qPCR. Mirids were allowed to feed for 3 days on empty vector (EV) and transformed plants silenced in the expression of: JA biosynthesis (ir*AOC*), trypsin proteinase inhibitors (ir*PI*) and diterpene glycosides (ir*GGPPS*). Statistical significance compared to control (EV) was analyzed by ANOVA followed by *post-hoc* Dunnett test, *n* = 3

We then used RT-qPCR to assess the expression of the candidate resistance genes in mirids feeding on three transgenic *N. attenuata* lines: plants deficient in all JA-inducible defenses (ir*AOC*) or plants deficient in particular groups of defenses, namely ir*GGPPS* (which does not produce DTGs) and ir*PI* (which does not produce TPIs) (Fig. 1). Our results show that, after three days of feeding, a more pronounced down-regulation was always observed in mirids feeding on plants with the larger number of silenced defenses (ir*AOC*) (Fig. 5). Although we could observe some decrease for some transcripts (as for some P450s) in mirids feeding on ir*PI* or ir*GGPPS* plants, such decreases were not statistically different from controls except in one instance. In contrast, a clear reduction in abundance of transcripts for four P450s, the GST and the UGT was observed in mirids feeding on ir*AOC* plants, and in these cases, the down-regulation estimated by RT-qPCR was comparable with the RNAseq estimated fold-change (Fig. 4b). RT-qPCR could not confirm a down-regulation of any serine protease or in the cytochrome *Tn*_P450_4 in mirids feeding on *ir*AOC plants (Fig. 4c).

### Detoxification enzymes in *T. notatus* transcriptome

#### CYP450 family

**Fig. 6:**
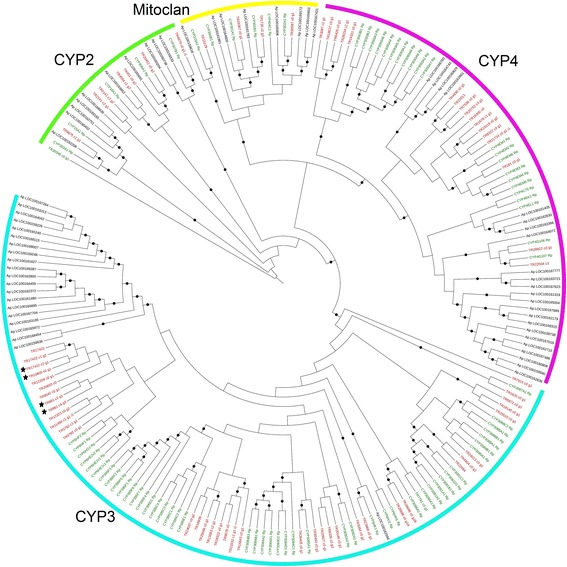
Phylogenetic relationships of *Tupiocoris notatus, Acyrthosiphon pisum* and *Rhodnius prolixus* P450s*.* Maximum-likelihood tree was built in Mega 6 based on MUSCLE amino acid alignment. Black dots indicate branch support >70 bootstrap. Black stars indicate *T. notatus* P450s down-regulated in mirids feeding on *ir*AOC plants. Genes from *A. pisum* (*black*) were taken from [53] and genes from *R. prolixus* (*green*) from [54]. *T. notatus* contigs are in red

*T. notatus* transcriptome was screened for the presence of P450 sequences. After manually clustering and merging, 68 PUTs were annotated as P450 by Blastx, 41 of which encoded full-length proteins. A ML tree was constructed from a multiple sequence alignment using P450 proteins from *T. notatus*, *A. pisum* and *R. prolixus* proteomes. Results show that *T. notatus* P450s are related to members of the four classical clades of the P450 family: CYP1, CYP2 and CYP3 clades and mitoclan (Fig. 6). In particular, *T. notatus* clade CYP3 was particularly enriched, followed by the CYP4 clade. All four PUTs annotated as P450 that were down-regulated during feeding on ir*AOC* plants belong to CYP3 clade (Fig. 6, Additional file 2: Figure S3).

#### Glutathione-s-transferase family

**Fig. 7:**
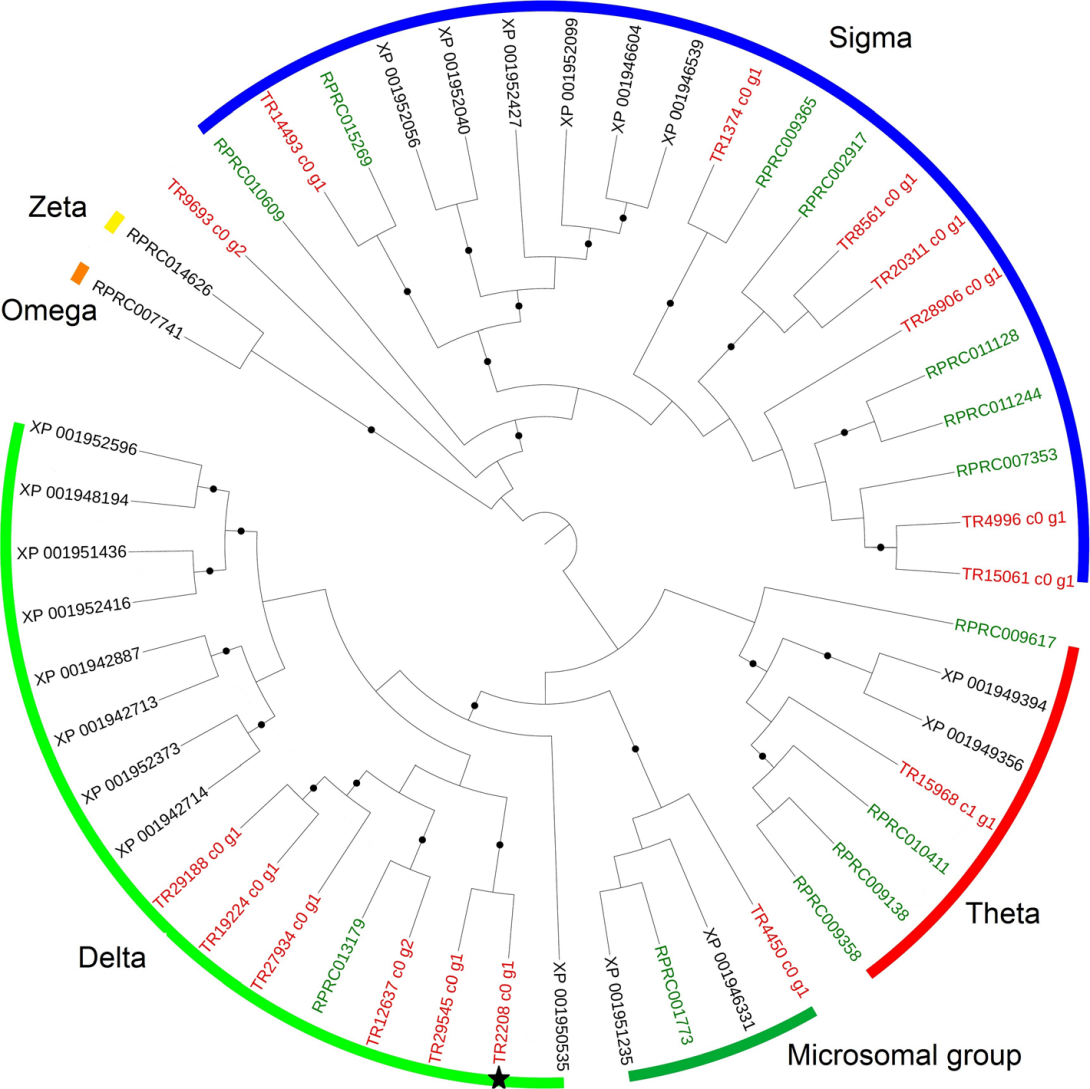
Phylogenetic relationships of *Tupiocoris notatus, Acyrthosiphon pisum* and *Rhodnius prolixus* GSTs*.* Maximum-likelihood tree was built in Mega 6 based on MUSCLE amino acid alignment. Black dots indicate branch support >70 bootstrap. Black star indicates the *T. notatus* GST PUT down-regulated in mirids feeding on *ir*AOC plants. Genes from *A. pisum* (*black*) were taken from [53] and genes from *R. prolixus* (*green*) from [54]. *T. notatus* contigs are in red

Sixteen PUTs coding for GST were identified (Fig. 7): fourteen contain a complete CDS whereas two were incomplete. They belong to three cytosolic classes (delta, sigma, and theta) and to the microsomal group; apparently two cytosolic groups are missing (zeta and omega). One PUT was not clearly classified. The classes with the largest number of members were sigma (7) and delta (6) whereas only a single PUT could be assigned to each of the other two classes. The delta class contained the PUT whose expression was down-regulated in *T. notatus* feeding on ir*AOC* plants (Fig. 7).

#### UGT-glycosyltransferase family

**Fig. 8:**
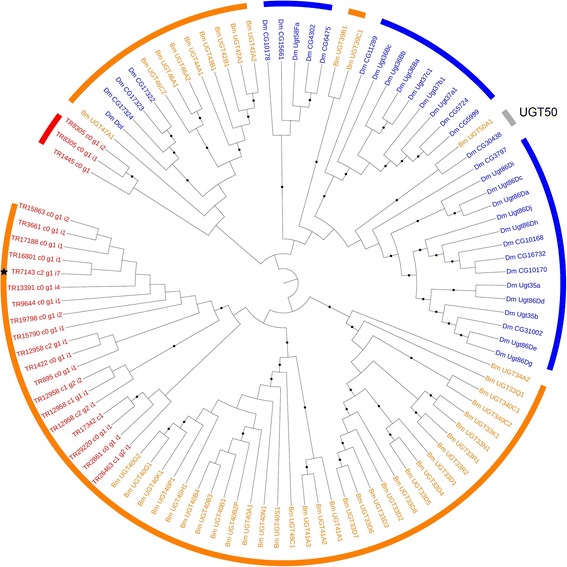
Phylogenetic relationships of *Tupiocoris notatus, Drosophila melanogaster* and *Bombyx mori* UGTs*.* Maximum-likelihood tree was built in Mega 6 based on MUSCLE amino acid alignment. Black dots indicate branch support >70 bootstrap. Black star indicates the *T. notatus* UGT PUT down-regulated in mirids feeding on *ir*AOC plants. Genes from *B. mori* (*orange*) were taken from [19] and genes from *D. melanogaster* (*blue*) from [55]. *T. notatus* contigs are in red

Twenty two PUTs encoding UGTs were identified, and fourteen contained a complete CDS. Phylogenetic analysis revealed that *T. notatus* UGTs clustered in a species-specific manner, according to the lineage-specific expansions that characterizes UGT family evolution in insects [19]. Notably, UGT50, a conserved UGT found in all insect genomes screened so far but missing from the *A. pisum* genome, is not present in the *T. notatus* transcriptome (Fig. 8).

## Discussion

Insect feeding induces a plant response, which often involves the accumulation of different toxic or anti-digestive metabolites to deter the attacker [1]. Such plant defense compounds usually elicit a counter-response in the insects, and for many insect species, it is thought that this counter-response consists of the activation of detoxification enzymes or processes that allow for the sequestration of the metabolites, in ways that are frequently particular to the natural history of the herbivore [5]. Transcriptional surveys of the responses of phytophagous insects consuming their host plants can provide insights into the processes by which insects specialize on and detoxify otherwise well-defended plants. Such genome-wide approaches remain relatively scarce as most studies examine the effects of single toxic compounds, frequently with artificial diets, simply because the genomic tools are lacking to manipulate the pathways responsible for defense metabolite production for most host plants.

*N. attenuata* produces a wide array of noxious compounds during *T. notatus* attacks, from phenolamides like caffeoyl-putrescine, phenolic compounds like chlorogenic and crypto-chlorogenic acid to diterpene-glycosydes (DTGs) and the proteinaceous effectors like trypsin-proteinase inhibitors (TPIs) [35]. All of these defense metabolites or proteins are inducible and their production is mediated by a rapid phytohormone signaling, such as JA that accumulates within a few minutes of insect attack [39]. By using ir*AOC* plants which are deficient in JA biosynthesis due to the silencing of Na*AOC*, we were able to analyze in detail the global response of *T. notatus* to the entire suite of JA-elicited defense compounds produced by its host plant, and identify specific candidate genes which allow *T. notatus* to feed on *N. attenuata*. The results were consistent with the general expectation for specialist herbivores, whose adaptation to the host plant is thought to involve a small number of fine-tuned and highly efficient detoxification mechanisms. In contrast, generalist herbivores rely on a broader array of detoxification genes, with different degrees of substrate specificity and efficiency [5, 58]. Transcriptional rearrangements shown by *T. notatus* feeding on *ir*AOC plants are limited, with a greater number of down-regulated transcripts. A similar pattern has been found in a previous comparative study with another *N. attenuata* specialist, the lepidopteran *M. sexta*. Compared to the attack by the generalist *Heliothis virescens,* attack from *M. sexta* larvae mainly down-regulated its transcripts feeding on JA-silenced plants, whereas the generalist both up- and down- regulated a larger number of genes [58].

The results showed that among down-regulated genes, some of them were annotated as UGTs, GSTs and P450s. All of these enzymes are well-known as detoxification enzymes, which are able in some cases to even detoxify insecticides, providing resistance in the field [59]. After contig merging and validation by RT-qPCR, we identified six tentative unique genes (PUTs) from *T. notatus* corresponding to one UGT, one GST and four P450s whose expression decreased during feeding on JA-silenced plants. Classification provided by a further phylogenetic analysis were consistent with a role for these genes in detoxification; the *T. notatus* GST belongs to the delta class, which is thought to function together with members of the epsilon class in xenobiotic metabolism [60], and the four down-regulated *T. notatus* P450s belong to the CYP3 clan which has been linked to xenobiotic metabolism and insecticide resistance [6]. P450s, GSTs and UGTs usually function cooperatively and participate in two different phases of xenobiotic detoxification. P450 enzymes are thought to carry out a first detoxification step (phase I reactions), which increase the molecule’s hydrophilicity. Transferase enzymes, like GSTs of UGTs, participate in phase II reactions which usually act on the products of the phase I reactions, and add side groups, such as glucuronides, which further increase the compound’s water solubility for later excretion or sequestration. Genes encodings P450s, GSTs and UGTs implicated in the metabolism of plant toxins are thought to provide the foundation of the detoxification mechanisms characteristic of generalist insects that cope with a more diverse and unpredictable array of plant defenses [5]. In contrast, it is thought that highly adapted insects possess more specialized enzymes that enable them to convert specific toxic products from their host plants to less toxic ones, as in the case of glucosinolate tolerance in certain species of Lepidoptera [61]. The results suggest that *T. notatus* employs a cooperative detoxification system based on P450s, GSTs and UGTs to metabolize tobacco defense metabolites, in a process more similar to the expectations for a generalist insect, than for a specialist like *T. notatus*. However, the *T. notatus* host, *N. attenuata*, produces a wide array of toxic compounds and *T. notatus* also feeds on a restricted number of other Solanaceous plants, like *D. wrightii* [27] and may move among hosts during its life. Hence, during its lifetime, *T. notatus* likely needs a highly adaptable detoxification system able to cope with the diversity of metabolites that it’s commonly exposed to. A previous comparison of two related CYP6Bs, which were both induced by and metabolize furanocoumarins, revealed that the enzyme from the generalist *H. zea* had a much wider substrate spectrum than the enzymes from the specialist *P. polyxenes* [12]. We infer that functional specialization of *T. notatus* P450s may account for its oligophagy, but at the same time retain the functional versatility required for coping with the diversity of secondary metabolites produced by *N. attenuata* [35].

After *T. notatus* attack, *N. attenuata* also produces TPIs, which decrease insect growth by inhibiting gut proteases and potentially reducing the availability of essential amino acids [25]. *T. notatus* feeding on ir*AOC* down-regulated the expression of six different serine proteases, results consistent with the well-documented strategies for coping with diets high in PIs: overproduction of inhibitor-sensitive digestive proteases, expression of inhibitor-insensitive isoforms or activation of proteases that hydrolyze plant PI [26]. However, the results could not be confirmed by RT-qPCR, which showed no differences in serine protease expression between *T. notatus* feeding on EV or ir*AOC* plants (and also not in ir*PI* plants, which were specifically silenced in TPI expression). Although these discrepancies were not observed for the other down-regulated transcripts, the differences in serine protease expression might be explained by the use of different biological replicates for each technique. Also, discrepancies between the two methods used for estimating counts from read mapping may be the responsible for false positives. Down-regulated serine proteases were identified using count matrix estimated by RSEM but not by Corset (except for the contig TR15565_c1_g1 which was a chimera between transcripts encoding a serine-protease and a protein from endoplasmic reticulum). Overall, the results obtained with Corset tended to be more accurate than those obtained with RSEM. Corset identified contigs belonging to seven clusters that could be merged into six PUTs (one GST, one UGT, three P450s and a serine-protease), and RT-qPCR confirmed a down regulation for four of these PUTs (GST, UGT and two P450s). In contrast, results obtained using RSEM-estimated count matrix were confirmed by RT-qPCR only for three (three P450s) of ten PUTs (four P450s and six serine-proteases). In general, our whole-body RNAseq analysis yielded only a handful of candidate detoxification genes which were DE in *T. notatus* when feeding on *ir*AOC plants. This may be due to several reasons. The use of entire insects may have masked expression differences among specific tissues, such as the midgut which is commonly involved in detoxification.

Expression of PUTs encoding defensive enzymes, which were down-regulated in *T. notatus* feeding on JA-silenced ir*AOC* plants, was further tested in mirids feeding on a different *N. attenuata* transgenic line, which specifically does not produce DTGs. When mirids were allowed to feed on ir*GGPPS* plants, results revealed that down-regulation was always not as strong compared to mirids feeding on ir*AOC* plants, suggesting that the putative detoxification mechanisms mediated by CYP450s, UGT and GST was not specifically involved in DTG detoxification but probably tuned to other JA-mediated defense compounds. Another possibility is that the activation of these enzymes was elicited by oxilipin products, such as JA or its conjugates, which accumulate in EV and *ir*GGPPS but not in ir*AOC* plants during *T. notatus* attack. Indeed, it has been shown that insects can perceive phytohormones. *Helicoverpa zea* responds to the ingestion of large quantities of JA and salicylate by activating four P450 genes [62]. Consistent with this possibility is the observation that another hemipteran (*Empoasca* sp.) which occasionally feeds on *N. attenuata* plants in nature, prefers to feed on plants deficient in JA accumulation, independently of the major defensive compounds elicited by JA signaling [39].

## Conclusions

 Here we identify and further validate the accumulation of transcripts involved in the adaptation of *T. notatus* to its host plant, *N. attenuata*. The RNAseq approach identified six transcripts which were down-regulated in *T. notatus* feeding on JA-silenced *ir*AOC plants and which encode detoxification enzymes which are thought to function cooperatively to metabolize *N. attenuata’s* major defensive compounds. The actual function of these transcripts in *T. notatus* can be now rigorously tested with plant-mediated RNA silencing [63], which can be conducted in field experiments [21]. Thus, our results provide a first step in understanding the mechanisms by which *T. notatus* exploits *N. attenuata* as host.

## Additional files

Additional file 1: Table S1. Putatively unique transcripts (PUTs) used in real-time qPCR (RT-qPCR) expression analysis. (XLSX 12 kb)
Additional file 2:
**Figure S1.** Most represented KEGG pathways in T. notatus transcriptome. **Figure S2.** Outcomes of differential expression analysis run in EdgeR using count matrix estimated with Corset or RSEM. **Figure S3.** Heat map of expression values of T. notatus P450 PUTs. (PDF 628 kb)
Additional file 3: Table S2. Differentially expressed isotigs identified using RSEM estimated abundances. (XLS 40 kb)
Additional file 4: Table S3. Differentially expressed clusters identified using CORSET estimated abundances. (XLS 46 kb)
