# Supplementary material for: Transcriptome profiling reveals differential gene expression of detoxification enzymes in a hemimetabolous tobacco pest after feeding on jasmonate-silenced Nicotiana attenuata plants
Source: BMC Genomics. 2016 Dec 8;17:1005. doi: 10.1186/s12864-016-3348-0 (PMC5146904; doi:10.1186/s12864-016-3348-0)
Supplement: Additional file 2: — Figure S1. Most represented KEGG pathways in T. notatus transcriptome. Figure S2. Outcomes of differential expression analysis run in EdgeR using count matrix estimated with Corset or RSEM. Figure S3. Heat map of expression values of T. notatus P450 PUTs. (PDF 628 kb) [file 12864_2016_3348_MOESM2_ESM.pdf]

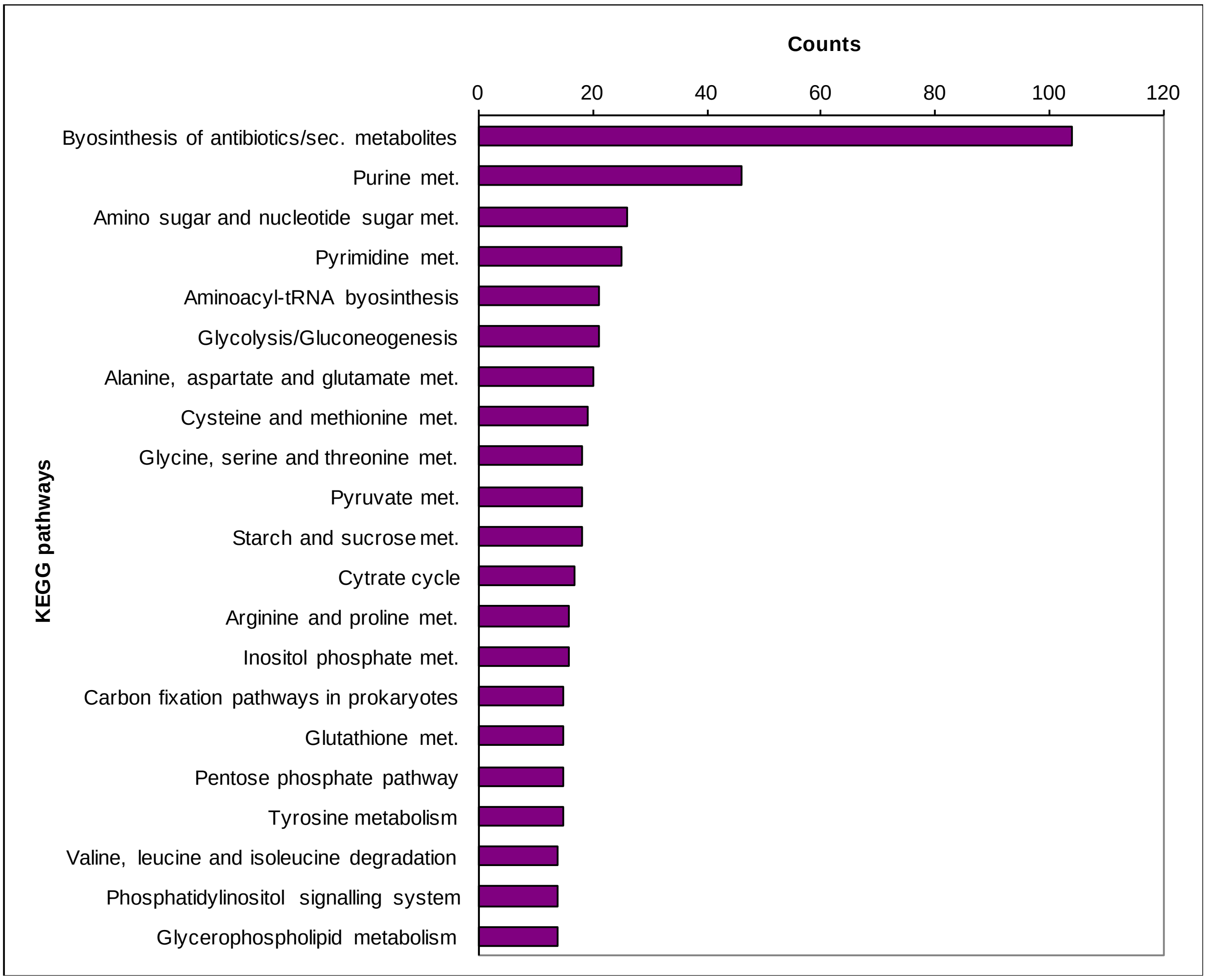

Figure S1. Most represented KEGG pathways in *T. notatus* transcriptome. Transcriptome annotation was performed with Blast2GO. Met. indicates metabolism.

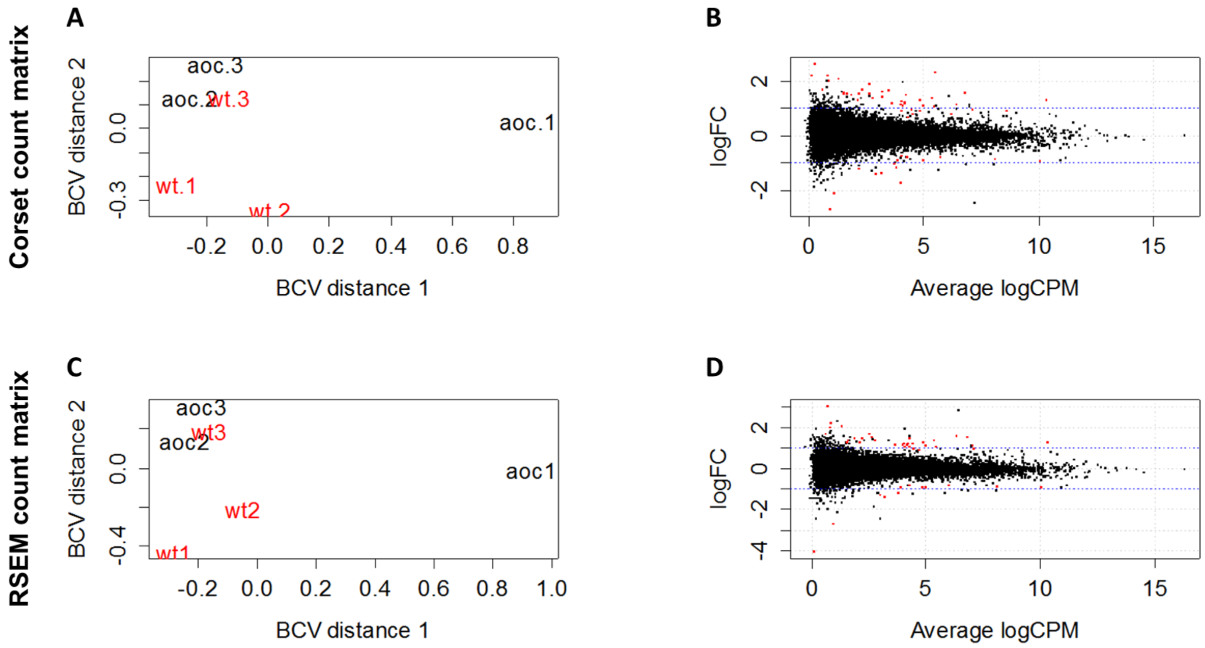

Figure S2. Outcomes of differential expression analysis run in EdgeR using count matrix estimated with Corset or RSEM. A) and C) multidimensional scaling (MDS) plots showing sample relations among replicates and treatmens. B) and D) Smearplots showing differentially expressed (DE) genes identified with EdgeR. Red spots highlit DE contigs at FDR < 0.05. Dotted blue lines indicate log fold-change (FC) 1. CPM stands for counts per million.

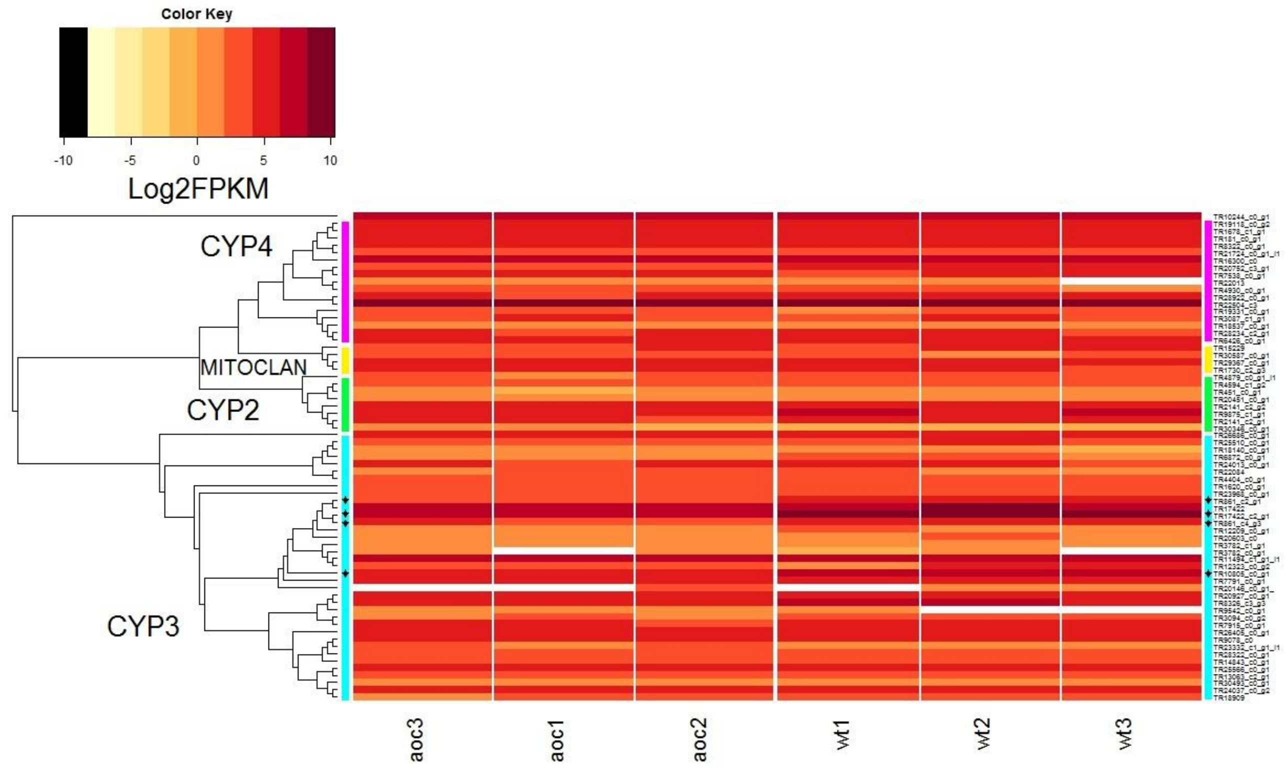

Figure S3. Heat map of expression values of *T. notatus* P450 PUTs. Values calculated with RSEM-estimated matrix abundances were used. Maximun likelihood tree calculated for all *T. notatus* P450 PUTs was used as row dendogram. Black stars highlitgh differentially expressed P450s. Classification of P450s based on Figure 6 (yellow = mitoclan, green = CYP2 clan, pink = CYP4 clan, light blue =CYP3 clan).
